# Psychometric properties of an Arabic translation of the shortest version of the Central Religiosity Scale (CRS-5) in a sample of young adults

**DOI:** 10.1186/s40359-023-01431-9

**Published:** 2023-11-18

**Authors:** Feten Fekih-Romdhane, Nathalie El Tawil, Christian-Joseph El Zouki, Karolina Jaalouk, Sahar Obeid, Souheil Hallit

**Affiliations:** 1 The Tunisian Center of Early Intervention in Psychosis, Department of Psychiatry “Ibn Omrane”, Razi Hospital, 2010 Manouba, Tunisia; 2 Faculty of Medicine of Tunis, Tunis El Manar University, Tunis, Tunisia; 3 Faculty of Arts and Sciences, Holy Spirit University of Kaslik, P.O. Box 446, Jounieh, Lebanon; 4 School of Medicine and Medical Sciences, Holy Spirit University of Kaslik, P.O. Box 446, Jounieh, Lebanon; 5 UFR de Médecine, Université de Picardie Jules Verne, 1 Rue des Louvels, Amiens, 80037 France; 6 Faculté d’Ingénierie et de Management de la Santé, 42 rue Ambroise Paré, Loos, 59120 France; 7 Faculty of Medicine, Lebanese University, Hadat, Lebanon; 8 Social and Education Sciences Department, School of Arts and Sciences, Lebanese American University, Jbeil, Lebanon; 9 Psychology Department, College of Humanities, Efat University, Jeddah, 21478 Saudi Arabia; 10 Applied Science Research Center, Applied Science Private University, Amman, Jordan; 11 Research Department, Psychiatric Hospital of the Cross, Jal Eddib, Lebanon

**Keywords:** Central religiosity scale, CRS-5, Arabic, Psychometric properties, Validation

## Abstract

**Background:**

There is a dearth of research on religiosity in Arabic-speaking populations, partly due to a lack of universal, standardized and valid instruments to assess this construct. We sought through this study to establish the psychometric properties of an Arabic translation of the shortest version of the Central Religiosity Scale (CRS-5), a widely used measure of religiosity that can be applicable to most religious traditions, thus allowing for worldwide cultural and trans-religious comparisons.

**Method:**

A total of 352 Lebanese young adults enrolled in this study with a mean age of 25.08 years (*SD* = 9.25) and 73.3% women. The forward-backward method was adopted to translate the original English version of the CRS-5 to Arabic.

**Results:**

We ran an Exploratory Factor Analysis for the CRS-5 to test whether the expected dimensionality is suitable for the subsequent Confirmatory Factor Analysis (CFA). The model found replicates the originally proposed five items and one-factor model. Our findings demonstrated that the Arabic CRS-5 achieved good levels of composite reliability, with a McDonald’s ω coefficient of .85. A multi-group CFA was modelled for the examination of measurement invariance of the Arabic CRS-5 across gender at the metric, configural, and scalar levels. Between-gender comparisons revealed no significant differences between males and females regarding CRS-5 scores. Finally, we found that religiosity was positively correlated with positive mental health aspects (i.e., social support) and inversely correlated with negative mental health aspects (i.e., suicidal ideation, depression, social anxiety and entrapment); thus attesting for the convergent validity of the CRS-5 as a measure of centrality of religiosity.

**Conclusion:**

Pending further validations with larger and more representative populations, we preliminarily suggest that the Arabic CRS-5 is psychometrically sound, and can be recommended for use for research and clinical purposes in Arabic-speaking people of various religions and cultures.

**Supplementary Information:**

The online version contains supplementary material available at 10.1186/s40359-023-01431-9.

## Introduction

Religiosity is commonly defined by the scientific community as “adherence to beliefs, doctrines, ethics, rituals, texts, traditions, and practices related to a higher power and associated with an organized group” [1]. Religion and religiosity (either intrinsic or extrinsic) are complex, multifaceted constructs that occupy a pivotal place in people’s lives in contemporary societies, and it substantially influences human thinking, behavior and decision-making [2]. Meta-analytic findings have consistently shown that, in various populations from different parts of the world, religiosity is closely linked to mental health and well-being [3–8]; with the vast majority of reported effects of religiosity on mental health being beneficial across religions, communities and countries. Consequently, religious interventions have been developed and widely tested through randomized clinical trials for their effectiveness in improving psychopathology symptoms. Findings have generally revealed that religious interventions have proven effective for reducing clinical symptoms (such as anxiety, stress, depression, and alcoholism) [9], promoting quality of life and well-being [10].

In Arab countries, religiosity represents an integral part of individuals’ daily lives (e.g., [11–13]). Psychopathology seems to be characterized by higher levels of religious content in Arab patients compared to those of Western origin (e.g., religious and blasphemous obsessions along with compulsive prayer-related washing and cleaning in patients with obsessive compulsive disorder [14–16]; religious and superstitious auditory hallucinations in patients with schizophrenia [17]). In Arab cultural backgrounds, religiosity was shown to inversely correlate with mental health symptoms (e.g., anxiety [18], depression [19], suicidality [20], substance use [21]), mental illness stigma [22]; and to positively correlate with favourable attitudes toward people with mental illnesses [23]. At the same time, a cross-cultural population-based study from 16 Arab countries and 10,036 individuals showed that endorsing religious causations of mental illness are linked to more negative attitudes toward help-seeking [24]. In addition, most Arab individuals have been reported to seek help from religious and traditional healers before any contact with mental health services (e.g., [25, 26]); which might result in substantial delays in mental health care [27]. This has led several researchers to call for culture- and religion-adapted assessment tools, psychological models, therapeutic interventions and techniques in Arabic-speaking settings (e.g., [28–30]).

Overall, there appears to be a complex interplay between religiosity and mental health determinants and indicators in the Arab world. Surprisingly, however, Arab populations remain highly under-researched in this field of study compared to English-speaking, Western, and Christian populations [31]. Additionally, several gaps and methodological shortcoming can be identified in the literature on religiosity and mental health-related topics in Arab populations. For instance, most of the prior research in this area has examined religious factors through self-developed measures or qualitative methods (e.g., [32–37]). Other authors relied on single-item measures to assess religiosity (e.g., [38]). However, this approach is problematic because its validity and reliability are debatable, and it does not enable to define precise criteria according to which participants produced their response [39]. To our knowledge, it is only recently that two religiosity scales have been developed and validated in the Arabic language. The first one is the Arabic religiosity scale [40], which has been specifically designed for Arab people and has not been translated/validated in other languages or cultures; thus precluding any comparisons across groups of different religious, ethnic, and cultural backgrounds. Another issue with this measure is that important psychometric properties have not been studied, such as Exploratory Factor Analysis (EFA)-to-Confirmatory Factor Analysis (CFA) strategy and measurement invariance. The second instrument is the Muslim Belief into Action (BIAC) Scale; which is a relatively narrow measure focusing upon only one aspect of religiosity, i.e. the degree of translation of personal beliefs into real-life actions, and can only be used among Arabs of Muslim religion. However, while Arab communities comprise a majority of Muslims [41], Christianism is the second largest religion (with Arab Christians being estimated to be around 15 million people).

To address these major gaps and contribute to advancing the literature on this fundamental question, we propose in the present study to provide an Arabic validated version of the most widely used religiosity measure globally, i.e. the Centrality of Religiosity Scale (CRS). The CRS is a self-report measure developed by Huber [42–46] to assess the importance, salience, or centrality of religious meanings within an individual’s personality structure. The CRS has been used in more than 100 studies, 25 countries, and 100,000 individuals [39]. Six length versions of the CRS exist; all of them measure five core-dimensions of religiosity, i.e. intellection, ideological, public practice, private practice, and religious experience dimensions [39]. The Intellectual dimension refers to social expectations that religious individuals have some religious literacy. The Ideological dimension refers to social expectations that religious individuals have in terms of their beliefs and convictions of plausibility of the existence of a transcendent reality. The Public Practice involves patterns of actions through which religious individuals express their belonging to a specific religious community by public participation and sharing of collective practices and rituals. The Private Practice refers to the expectation that religious individuals engage in individualized religious rituals and activities which are practiced in the private space (e.g., prayers, meditation). Finally, the Religious Experience dimension involves the social expectation that religious individuals experience the feeling of being connected to, or in a direct contact with, an ultimate reality or something divine, which, in turn, emotionally affects them. Measuring the intensity and frequency of each of these dimensions reflects the extent to which all aspects of religiosity are central to one’s life. The five core-dimensions as approached by Huber [47] may be viewed as modes of activation of personal religious constructs, which occurs when the individual anticipates something with a religious meaning, and can, therefore, be applied independent of religious affiliation or any confessional bias. In this regard, Huber and Huber [39] explained in their original validation paper how the five core-dimensions (as reflected in the items’ content) can be “acceptable in most religious traditions allowing for trans-religious generalization of the measure”. For example, the basic “belief in the existence of God or something divine”, or belonging to religious communities which is manifested in “taking part in religious services” are common to most religious traditions [39]. The original versions, designed for Abrahamic religions with a monotheistic concept of God, contain 15 (three per dimension) and 10 (two per dimension) items [42–44]. Three “interreligious” versions of the CRS (CRS-7, CRS-14, and CRS-20) have been developed to reflect their openness to polytheistic practices and concepts [39]. The briefest version is composed of five items (CRS-5), each of them assessing one of the five above-mentioned dimensions [39].

The latter “most economical version” showed adequate reliability and acceptable fit indexes [39]. The psychometric qualities of the CRS-5 have also been demonstrated in other linguistic versions, including Brazilian [48], Georgian [49], Chinese [50], Vietnamese [51], Portuguese [52], Romanian [53], Russian [54], Filipino [55], and Swedish [56]. However, none of the CRS versions have been translated to the Arabic language so far; and the reliability/validity of the CRS in the Arabic-speaking contexts are still to be demonstrated. In this regard, we sought through this study to establish the psychometric properties of an Arabic translation of the shortest version of the CRS in a sample of Arabic-speaking community adults from Lebanon. We hypothesized that: (1) a one-factor solution will provide a good fit to the data, (2) the Arabic CRS-5 will demonstrate high internal consistency and measurement invariance across gender groups, and (3) CRS-5 scores will show adequate patterns of correlations with related variables. In particular, we expect that higher CRS-5 score be positively correlated with social support; and negatively correlated with suicidal ideation, depression, social anxiety and entrapment constructs.

## Methods

### Participants

A total of 352 Lebanese young adults enrolled in this study with a mean age of 25.08 years (*SD* = 9.25) and 73.3% women. Characteristics of both split-half subsamples are displayed in Table 1.

**Table 1:** Sociodemographic characteristics of the participants

Variable	Total sample (***N*** = 352)	First split-half subsample (***n*** = 183)	Second split-half subsample ***(n*** = 169)
Gender			
Males	94 (26.7%)	48 (26.2%)	46 (27.2%)
Females	258 (73.3%)	135 (73.8%)	123 (72.8%)
Age (in years)	25.08 ± 9.25	24.25 ± 8.02	25.98 ± 10.37

### Measures

#### Demographics

Participants were asked to provide their demographic details consisting of age and gender.

#### The Central Religiosity Scale (CRS-5) [39]

The CRS-5 comprises five items measuring the following religiosity dimensions: (1) Intellect (i.e. “How often do you think about religious issues?”), (2) Ideology (i.e., “To what extent do you believe that God or something divine exists?”), (3) Public Practice (i.e., “How often do you take part in religious services?”), (4) Private Practice (i.e., How often do you pray?), and (5) Experience (i.e., “How often do you experience situations in which you have the feeling that God or something divine intervenes in your life?”). The five items have different verbal response anchors, and a “do not know” option. For the three dimensions Ideology, Intellect, and Religious Experience, items are rated on a five-point Likert scale from 1 to 5. For the Public Practice dimension, the item has a response modality ranging from 1 to six. The fifth item related to the Private Practice dimension; response modality varies from 1 to 8.

#### Entrapment Scale Short Form (E-SF)

This is a 4-item scale measured feelings of being trapped or stuck on a 5-point Likert scale [57]. Higher scores indicate a stronger sense of entrapment. We employed the Arabic version that has been validated in Lebanon [58].

#### Multidimensional scale of perceived social support

This 12-item scale [59], validated in Lebanon [60], measured perceived social support from family, friends and significant others. A 7-point Likert scale is used to grade each statement, with 1 representing very strong disagreement and 7 representing very strong agreement. Higher scores signify stronger perceived social support.

#### Patient Health Questionnaire (PHQ-9)

This widely used measure evaluates, screens for, and quantifies the degree of depression experienced during the past 2 weeks [61]. Validated in Lebanon [62], it comprises of 9 items, and the scores range from “0” (not at all) to “3” (almost daily) with higher scores implying more severe symptoms of depression. The items come as follows: “Feeling down, depressed or hopeless”, “Poor appetite or overeating” and “feeling tired or having little energy”.

#### Social Phobia Inventory (SPIN)

It is a 17-item inventory for the assessment of social anxiety disorder or social phobia manifestations [63]. Respondents filled several statements like “I am afraid of people in authority”, “I would do anything to avoid being criticized” and “I avoid talking to people I don’t know”. The scale, validated in Lebanon [64], measures each of the social anxiety disorder’s feature dimensions over the course of the previous week. Each response ranges from 0 (not at all) to 4 (extremely) on a 5-point Likert scale. Higher scores represent greater social anxiety.

#### The Columbia-Suicide Severity Rating Scale (C-SSRS)

This 6-item tool, validated in Lebanon [65, 66], is used to assess suicidal ideation and behavior. A score of 0 indicates the absence of suicidal ideation, while a score of 1 or more confirms the opposite [67].

#### Translation procedure

The CRS-5 was converted from English to Arabic by a mental health specialist and then from Arabic to English by a second certified translator. Upon completion of this step, the translators compared the English versions to determine whether the variables had the same value. A pilot study was conducted on 30 persons to make sure all questions were well understood; consequently, no considerable differences were found for the CRS-5. The English and Arabic versions of the CRS-5 are included as Supplementary files.

### Procedures

Ethics approval for this study was obtained from the Psychiatric Hospital of the Cross ethics committee (HPC-040-2022). All data were collected via a Google Form link, between November 2022 and January 2023. The project was advertised on social media and included an estimated duration. Inclusion criteria for participation included being of a resident and citizen of Lebanon, with an age between 18 and 29 years. Internet protocol (IP) addresses were examined to ensure that no participant took the survey more than once. After providing digital informed consent, participants were asked to complete the instruments described above, which were presented in a pre-randomised order to control for order effects. The survey was anonymous and participants completed the survey voluntarily and without remuneration.

### Analytic strategy

#### Data treatment

There were no missing responses in the dataset. To examine the factor structure of the CRS-5 scale, we used an EFA-to-CFA strategy [68]. To ensure adequate sample sizes for both EFA and CFA, we split the main sample using an SPSS computer-generated random technique; sample characteristics of the two split-halves are reported in Table 1. There were no significant differences between the two subsamples in terms of mean age, *t*(350) = 1.753, *p* = .084 and gender, χ^2^(1) = .044, *p* = .834.

#### Exploratory factor analysis

To explore the factor structure of CRS-5 scale, we computed a principal component analysis with the first split-half subsample using the FACTOR software [69, 70]. We verified all requirements related to item-communality [71], average item correlations, and item-total correlations [72]. The Kaiser-Meyer-Olkin (KMO) measure of sampling adequacy and Bartlett’s test of sphericity ensured the adequacy of our sample [73]. The procedure for determining the number of factors to extract was parallel analysis (PA [74]; using the Pearson correlation matrix. Weighted Root Mean Square Residual (WRMR) was also calculated to assess the model fit (values < 1 have been recommended to represent good fit [75];.

Item retention was based on the recommendation that items with “fair” loadings and above (i.e., ≥ .33) and with low inter-item correlations (suggestive of low item redundancy [76].

#### Confirmatory factor analysis

We used data from the second split-half to conduct a CFA of the model obtained in the EFA, using the SPSS AMOS v.29 software. A previous study suggested that the minimum sample size to conduct a confirmatory factor analysis ranges from 3 to 20 times the number of the scale’s variables [77]. Therefore, we assumed a minimum sample of 100 participants needed to have enough statistical power based on a ratio of 20 participants per one item of the scale, which was exceeded in this subsample. Parameter estimates were obtained using the robust maximum likelihood method and fit indices. Additionally, evidence of convergent validity was assessed in this subsample using the average variance extracted (AVE), with values of ≥ .50 considered adequate [78].

#### Gender invariance

To examine gender invariance of CRS scores, we conducted multi-group CFA [79] using the second split-half subsample. Measurement invariance was assessed at the configural, metric, and scalar levels [80]. Configural invariance implies that the latent CRS-5 variable(s) and the pattern of loadings of the latent variable(s) on indicators are similar across gender (i.e., the unconstrained latent model should fit the data well in both groups). Metric invariance implies that the magnitude of the loadings is similar across gender; this is tested by comparing two nested models consisting of a baseline model and an invariance model. Lastly, scalar invariance implies that both the item loadings and item intercepts are similar across gender and is examined using the same nested-model comparison strategy as with metric invariance [79]. Following previous recommendations [79, 81], we accepted ΔCFI ≤ .010 and ΔRMSEA ≤ .015 or ΔSRMR ≤ .010 (.030 for factorial invariance) as evidence of invariance. We aimed to test for gender differences on latent CRS-5 scores using an independent-samples *t*-test only if scalar or partial scalar invariance were established.

#### Further analyses

Composite reliability in both subsamples was assessed using McDonald’s ω and its associated 95% CI, with values greater than .70 reflecting adequate composite reliability [82]. McDonald’s ω was selected as a measure of composite reliability because of known problems with the use of Cronbach’s α [83]. To assess convergent and criterion-related validity, we examined bivariate correlations between CRS-5 scores and those on the additional measures included in the survey (suicidal ideation, entrapment, depression, social anxiety, and social support) using the total sample. All scores had normal distribution, as identified by skewness and kurtosis values varying between ±1.96 [84]; therefore, Pearson correlation test was used. Based on [85], values ≤ .10 were considered weak, ~ .30 were considered moderate, and ~ .50 were considered strong correlations.

## Results

### Exploratory factor analysis

For the first split-half subsample, Bartlett’s test of sphericity, *χ*^2^(10) = 428.3, *p* < .001, and KMO (.835) indicated that the CRS items had adequate common variance for factor analysis. The results of the EFA revealed one factor, which explained 64.19% of the common variance (item-factor loadings ≥ .80). The WRMR value was also adequate (.115; 95% CI .088–.135), indicating good fit of the model. The factor loadings are reported in Table 2.

**Table 2:** Items of the CRS-5 scale in English and factor loadings derived from the Exploratory Factor Analyses (EFA) in the first split-half subsample, and standardised estimates of factor loadings from the Confirmatory Factor Analysis (CFA) in the second split-half subsample

	EFA	CFA
Item		
1. Intellect - How often do you think about religious issues?	.66	.35
2. Ideology - To what extent do you believe that God or something divine exists?	.87	.83
3. Public practice - How often do you take part in religious services?	.76	.64
4. Private practice - How often do you pray?	.86	.86
5. Experience - How often do you experience situations in which you have the feeling that God or something divine intervenes in your life?	.83	.79

#### Factor structure congruence and composite reliability

McDonald’s ω was adequate in women (*ω* = .80), men (*ω* = .89), and the total subsample (*ω* = .85).

### Confirmatory factor analysis

CFA with the second split-half subsample indicated that fit of the unidimensional model of CRS-5 scale was generally acceptable: χ^2^/df = 13.36/5 = 2.67, RMSEA = .10 (90% CI .036, .166), SRMR = .036, CFI = .975, TLI = .951. The standardised estimates of factor loadings were all adequate (see Table 2). The convergent validity for this model was adequate, as AVE = .52.

#### Composite reliability

Composite reliability of scores was adequate in women (*ω* = .84), men (*ω* = .87), and the total sample (*ω* = .82).

### Gender invariance

Next, we tested for gender invariance based on the unidimensional model of *CRS-5*scores in the second split-half subsample. No significant difference was found in terms of *CRS-5*scores between women (*M* = 17.80, *SD* = 4.32) and men (*M* = 19.01, *SD* = 4.17) in the second subsample, *t*(167) = 1.654, *p* = .100, *d* = .284 (Table 3).

**Table 3:** Measurement invariance across gender in the second split-half subsample

Model	χ^2^	*df*	CFI	RMSEA	SRMR	Model Comparison	Δχ^2^	ΔCFI	ΔRMSEA	ΔSRMR	Δ*df*	*p*
Configural	20.65	10	.970	.080	.064							
Metric	27.26	14	.962	.075	.092	Configural vs metric	6.61	.012	.015	.028	4	.158
Scalar	30.00	18	.966	.063	.093	Metric vs scalar	2.74	.004	.012	.001	4	.602

*CFI* Comparative fit index, *RMSEA* Steiger-Lind root mean square error of approximation, *SRMR* Standardised root mean square residual

### Concurrent validity

CRS-5 was negatively and significantly correlated with suicidal ideation, entrapment, depression and social anxiety, but positively and significantly associated with social support (Table 4).

**Table 4:** Bivariate correlations between CRS-5 score and other measures included in the study and age

	1	2	3	4	5	6	7
1. CRS-5	1						
2. Suicidal ideation	−.27***	1					
3. Entrapment	−.21***	.56***	1				
4. Depression	−.18**	.55***	.71***	1			
5. Social anxiety	−.18**	.35***	.46***	.46***	1		
6. Social support	.14**	−.37***	−.39***	−.37***	−.27***	1	
7. Age	−.02	−.100	−.09	−.18**	−.22***	−.05	1

**p < .01, ***p < .001

## Discussion

This study was motivated by a dearth of research on religiosity in Arabic-speaking populations, partly due to a lack of universal, standardized and valid instruments to assess this construct. The use of diverse measures of religiosity in international studies might lead researchers to different conclusions, retard scientific progress in the study of the relationship religiosity-aspects of mental health, and hamper clinicians’ efforts to understand the role of religiosity as a complementary treatment in mental health care [9]. Therefore, there appears to be a strong need for validating an Arabic version of the CRS, a measure of religiosity that can be applicable to most religious traditions, thus allowing for worldwide cultural and trans-religious comparisons [39]. Our findings provided support to the unidimensional structure of the Arabic CRS-5, its adequate composite reliability, its cross-gender invariance, and its appropriate convergent and Criterion-Related validity as attested by inverse correlations with negative mental health indicators.

We run an EFA for the CRS-5 to test whether the expected dimensionality is suitable for the subsequent CFA. The model found replicates the originally proposed five items and one-factor model of Huber and Huber (Intellect, Ideology, Public Practice, Private Practice, and Religious Experience) [39]. Consistent with our results, validation studies in other populations and cultural contexts (e.g., Russia [54], Portugal [52]) also found that the CRS-5 presented good fit indices for the unidimensional factor model. Some studies tested the dimensionality of different versions of the CRS and found mixed results. For instance, Esperandio [48] showed that a five-factor solution of the 10-item version presented better fit indices than the one-factor solution of the five-item version in a Brazilian sample. In contrast, Lee and Kuang [50] found that the single-factor solution of the CRS-5 indicated better fit indices than both the seven-item and the 15-item versions in a Chinese population. Ackert et al. [49] revealed that the CRS-5 had a comparable model fit to the 7-item version of the scale, and suggested that it can be suitable as a shorter alternative for interreligious studies when needed. Overall, evidence tend to support that the CRS-5 presents conventional and acceptable indices, making it the simplest and fastest measure of religiosity to use in different clinical and research settings all over the world.

Our findings demonstrated that the Arabic CRS-5 achieved good levels of composite reliability, with a McDonald’s ω coefficient of .85. This is in agreement with previous findings from the original validation work, which showed that the CRS-5 had good internal consistency with a Cronbach’s alpha coefficient of 0.85) [39]. Other linguistic versions of the CRS-5 validated in different countries also showed high reliability coefficients as evidenced by appropriate Cronbach’s alphas (e.g., Russian, *α* = 0.85 [54]; Chinese, *α* = 0.917 [50]; Brazilian, *α* = 0.852 [48]; Romanian Orthodox group *α* = 0.85, Romanian Pentecostal group *α* = 0.78 [53]; Vietnamese *α* = 0.85 [51]; Filipino *α* = .75 [55]; Swedish *α* = 0.92 [86]). Beyond reliability, a multi-group CFA was modelled for the examination of measurement invariance of the Arabic CRS-5 across gender at the metric, configural, and scalar levels. Findings ascertained the consistency of its measurement quality across gender groups. This suggests that all five items were understood in a similar manner by males and females, and that the scale can be used for gender comparisons in future research among Arabic-speaking populations. Our analyses yielded no significant gender differences in CRS-5 scores. Consistent with our findings, men and women showed no difference in their religiosity scores as assessed using CRS-5 in previous studies from different countries and contexts (e.g., Portugal [52], Brazil [48]). Contrarily, others studies found significant gender differences, with either females displaying higher CRS-5 scores (e.g., China [50]), or males having slightly higher mean scores (e.g., Philippines [55]). Although it has always been agreed in the sociology of religion that women exhibit higher levels of religiosity than men; recent research has shown that these large or universal differences between men and women may vary across cultures [87]. A large-scale multi-country study revealed that gender equality across cultures (i.e. the extent to which men and women share equal responsibilities, rights, and opportunities in society) negatively and consistently predicted religiosity levels (i.e. reported importance of God, frequency of prayer, and religious attendance) among men more than among women, resulting in a wider gender difference in religiousness in more gender-equal countries [88]. To explain their results, authors suggested that religion may be more appealing for men from less gender-equal cultures (such as Arab countries [89]) as it may afford more reproductive benefits (e.g., proscribing sexual promiscuity, modesty norms) and be more useful as a tool of social influence [88]. These suggestions, along with our present findings, concur with those of previous studies performed in Arab countries and populations and showing no gender differences in religiosity levels (e.g., [20, 90]). These cultural considerations could explain the reasons why men exhibited comparable mean CRS-5 scores as women in our sample.

Finally, our findings showed expected patterns of correlations between CRS-5 scores and study variables. Specifically, religiosity was positively correlated with positive mental health aspects (i.e., social support) and inversely correlated with negative mental health aspects (i.e., suicidal ideation, depression, social anxiety and entrapment). These findings are in line with previous observations suggesting that Arabs (either Muslims or Christians) hold strong religious convictions that potentially promote their mental and emotional health both in general and in crisis situations [91]. In particular, prior Arab studies have shown that religiosity was protective against suicidality [20], depression [19], and anxiety [18]. More religious Arab people have also been found to report higher levels of support from relatives and friends, as well as more frequent and intense interpersonal contacts (e.g., [92]). Similarly, previous studies performed among Turkish university students found that religiosity was associated with greater life satisfaction [93], as well as lower levels of depression, hopelessness [94] and loneliness [95]. Another study found that religiosity showed positive correlations with prosociality and satisfaction with life, and inverse correlations with anxiety among Turkish Muslims [96]. In sum, these results support the convergent validity of the CRS-5 as a measure of centrality of religiosity.

### Study limitations

This study is not without limitations. First, the sample was collected using an online convenience sampling, which is a non-probability sampling method that could lead to sampling bias. Second, participants were from one Arab country and culture (Lebanon), which prevents any generalization of the results to other Arab populations or countries. As such, future studies still need to confirm the robustness of the Arabic CRS-5 in other communities and cultural contexts. In addition, other psychometric properties have not been examined in the context of the present study, such as test-retest and inter-rater reliability. Finally, some characteristics have not been considered in this study such as religion, or mental state of the participants. Hence, future studies are recommended to diagnose the mental disorders of the study participants.

## Conclusion

Our goal was to provide a short, simple, and easy-to-use measure of religiosity for the broad Arabic-speaking communities in Arab countries and abroad. Findings provide support to the unidimensionality, reliability, gender invariance, and validity of the Arabic version of the CRS-5 in a sample of Lebanese community adults. Pending further validations with larger and more representative populations, we preliminarily suggest that the Arabic CRS-5 is psychometrically sound, and can be recommended for use for research and clinical purposes in Arabic-speaking people of various religions and cultures.

### Supplementary Information

**Additional file 1.**

## Data Availability

All data generated or analyzed during this study are not publicly available due the restrictions by the ethics committee (data are owned by the Psychiatric Hospital of the Cross). The dataset supporting the conclusions is available upon request to Ms. Rana Nader (rnader@naderlawoffice.com), a member of the ethics committee at the Psychiatric Hospital of the Cross.
