# Supplementary material for: Psychometric properties of an Arabic translation of the shortest version of the Central Religiosity Scale (CRS-5) in a sample of young adults
Source: BMC Psychol. 2023 Nov 18;11:400. doi: 10.1186/s40359-023-01431-9 (PMC10657561; doi:10.1186/s40359-023-01431-9)
Supplement: Supplementary file 1 — Additional file 1. [file 40359_2023_1431_MOESM1_ESM.pdf]

Table S1. Items of Central Religiosity Scale (CRS-5)

Items

1. How often do you think about religious issues?
2. To what extent do you believe that God or something divine exists?
3. How often do you take part in religious services?
4. How often do you pray?
5. How often do you experience situations in which you have the feeling that God or something divine intervenes in
